# Surface Modification of Attapulgite by Grafting Cationic Polymers for Treating Dye Wastewaters

**DOI:** 10.3390/ma14040792

**Published:** 2021-02-07

**Authors:** Huan Guo, Kai Xia, Mingzhao Cao, Xiaodong Zhang

**Affiliations:** 1 College of Chemistry and Chemical Engineering, Qingdao University, Qingdao 266071, China; hhuan0000@163.com (H.G.); xiakaiqd@163.com (K.X.); 2 Technological Research and Development Department, Shandong Tiexiong Xinsha Energy Co., Ltd., Heze 274916, China; caomingzhao880311@163.com

**Keywords:** modified attapulgite, dye adsorption, attapulgite clay, cationic polymer

## Abstract

In this study, the cationic polymer poly-epichlorohydrin-dimethylamine was immobilized on natural attapulgite to improve the dye adsorption capacities. Fourier transform infrared spectroscopy (FTIR), X-ray diffraction, nitrogen adsorption-desorption isotherms, scanning electron microscope (SEM) analysis, zeta potential analysis, and particle size analysis were used to determine the characteristics of modified attapulgite. Results showed that the poly-epichlorohydrin-dimethylamine had been successfully grafted onto the surface of attapulgite without altering its crystal structure. After cationic modification, the specific surface area of attapulgite obviously decreased, and its surface zeta potentials possessed positive values in the pH range from 3 to 11. The cation-modified attapulgite displayed high adsorption capacities for anionic dyes, and its maximum adsorption capacities were 237.4 mg/g for Reactive Black 5 and 228.3 mg/g for Reactive Red 239; this is corroborated by Langmuir’s isotherm studies. It was demonstrated that the two reactive dyes could be 100% removed from effluents when cation-modified attapulgite was used in column operation modes. Its treatment capacities were more than three times larger than that of activated carbon. The regeneration study verified better utilization and stability of the fabricated adsorbent in column operation. This work has conclusively confirmed the potential of the new modified attapulgite for effectively treating dye wastewaters.

## 1. Introduction

Dye wastewater is of increasing concern, due to the negative effects on public health and the environment [1,2]. The adsorption method is the most commonly used decolorization technology in wastewater treatment, and it has the advantages of simple operation, low cost, and excellent removal effects on almost any dye [3,4,5]. To date, different types of micro- and mesoporous adsorbents have been reported for removing dyes from aqueous solutions, including activated carbon [6], minerals [7], agricultural wastes [8], ion exchange resins [9,10], and organic/inorganic composites [11]. Nevertheless, many absorbing materials have the disadvantages of lower adsorption performance or higher price. The development of novel dye adsorbents with high efficiency, low cost and a wide range of sources has been a hot topic in the industry of decolorization of colored wastewaters.

Attapulgite (ATP) is a cheap and resource-rich natural hydrated magnesium aluminum silicate clay mineral [12]. Owing to its desirable properties—such as a large specific surface area and micro- and mesoporous structure, ATP is widely used as an adsorbent in various industries [12,13,14]. Many investigations have reported on the use ATP for the treatment of dye wastewaters, including cationic dyes (methylene blue, crystal violet) [15,16,17], reactive dyes (Reactive Red MF-3B, Reactive Blue KE-R, Reactive Black GR) [18,19], acid dyes (Acid Orange 7, Congo red) [20,21], and others. However, the dye adsorption capacity of natural ATP is relatively low compared to the equally commonly used activated carbon. Additionally, natural ATP has negative charges on its surface [12,18], resulting in inferior adsorption for anionic dyes. Researchers have reported some cationic surfactants that can be used to improve the selectivity and adsorption capacity of ATP by impregnation technique; these include octodecyl trimethyl ammonium chloride and hexadecyl trimethyl ammonium bromide [18,22,23]. However, the literature’s reports on the modification of ATP by cationic polymer through covalent bond are scarce. Poly-epichlorohydrin-dimethylamine (EPI-DMA) is a water-soluble cationic polyelectrolyte with ammonium ion. Previous studies have demonstrated that EPI-DMA can remove colloid particles and suspended solids from wastewaters through charge neutralization and adsorption bridging [24,25]. Additionally, it has been applied in anionic dye wastewater treatment as an effective flocculating agent, due to the high positive charge [26,27,28]; however, its good water solubility also limits its application in adsorption technology.

In this work, the cation-modified attapulgite (CM-ATP) was prepared by grafting the cationic polymer EPI-DMA onto natural ATP for the purpose of making full use of the adsorption abilities of ATP, as well as the decolorizing properties of EPI-DMA. The characteristics of CM-ATP were investigated in detail, and batch and column adsorption experiments were conducted.

## 2. Materials and Methods

### 2.1. Materials

Natural attapulgite (ATP) was supplied by Jiangsu Autobang International Co., Ltd. (Huaian, China). Its main chemical compositions (wt.%) are: SiO_2_, 44.93%; MgO, 21.52%; CaO, 23.67%; Al_2_O_3_, 3.86%; Fe_2_O_3_, 2.98%; K_2_O, 1.16%. C.I. Reactive Black 5 (RB5) and C.I. Reactive Red 239 (RR239) were obtained from BASF SE (Ludwigshafen, Germany), and Table S1 shows their chemical structures. Polyepichlorohydrin-dimethylamine (EPI-DMA) with cationicity of 4.92 mmol/g was prepared according to the technique in our previous report [29]. Commercial activated carbon (AC), with—methylene blue number of 195.5 mg/g—and other chemicals were procured from Shanghai Sinopharm Chemical Reagent Co., Ltd. (Shanghai, China).

### 2.2. Synthesis of Cation-Modified Attapulgite

Cation-modified attapulgite was synthesized in two steps (see Scheme S1). First, the 3-aminopropyltriethoxysilane (APTES) and natural ATP were used to prepare APTES-modified ATP (AM-ATP), based on the technique of Xue et al. [19]. Second, 20.0 g of AM-ATP was added into 15.0 g of EPI-DMA aqueous solution (20.0 wt.%, pH = 11) and stirred at room temperature. The above mixture then reacted in an oven at 50 °C for 24 h. It was subsequently purified through extraction with distilled water and dried at 50 °C. Thus, the cation-modified attapulgite (CM-ATP) was obtained. The cationic polymer content of CM-ATP was determined by the method of Yue et al. [30].

### 2.3. Characterization of Adsorbents

Scanning electron microscopy (JSM 7001F, Tokyo, Japan) was used for observing the microstructure of samples. The Fourier transform infrared spectroscopy (FTIR) spectra of samples were measured by an FTIR spectrophotometer (Nicolet IS-10, Madison, WI., USA). The particle size distribution of natural ATP and CM-ATP was measured by a laser particle size analyzer (RISE-2028, Jinan, China). The crystal structures of samples were analyzed by an X-ray diffractometer (DX2700, Dandong, China). A specific surface area analyzer (JW-BK200A, Beijing, China) was used for determining the specific surface area and pore diameter distribution of samples. A Malvern Nano ZS Zeta potentiometer (Malvern, UK) was used to analyze the surface potential of ATP before and after modification.

### 2.4. Equilibrium Adsorption of Dyes

Equilibrium adsorption experiments were conducted in Erlenmeyer flasks with batch procedures. The dye aqueous solutions were set at 500 mg/L, and the adsorbent dosage was 2 g/L. The effects of solution pH (3–11), salt (NaCl, 1–4 g/L), surfactant (dodecyltrimethylammonium chloride (DTAC), 1 g/L) and temperature (293–333 K) on adsorption were studied. Each sample was shaken for 2 h. The final solutions were determined by a UV-VIS spectrophotometer (Shimadzu, Kyoto, Japan), and their dye concentrations were calculated according to the standard curves. The percentages of dye removal and adsorption capacity were calculated according to the formulas in Table S2. Kinetic studies were performed at room temperature, and the concentration of dye solutions and adsorbent dosage were 500 mg/L and 2 g/L, respectively. The adsorption isotherm studies were carried out at room temperature with the initial dye concentration ranging from 100 to 500 mg/L.

The equations for calculating the thermodynamic parameters, kinetics models, and isotherm models used in this study are shown in Table S2.

### 2.5. Column Adsorption Experiments

Column adsorption experiments were carried out in an organic glass column that connected to a UV-VIS spectrophotometer [31]. The natural ATP, CM-ATP and commercial activated carbon were used as the column adsorbent. The bed height and flow rate of dye solutions (500 mg/L) were set as 2 cm and 2 mL/min, respectively. The solutions leaving the column were detected by the spectrophotometer at set time intervals to judge whether they contained dyes. The treatment capacity of the adsorbent in the column adsorption process was calculated using the formula below, Equation (1).

(1) $$Q=\frac{C\times V}{1000M}$$

where *Q* (mg/g), *C* (mg/L), *M* (g), and *V* (mL) are the treatment capacity in the column adsorption process, the dye solution concentration, the adsorbent mass, and the volume of colorless effluent, respectively.

The used CM-ATP was regenerated directly in the column. DTAC solution with a concentration of 0.05 mol/L and pH value of 12.5 was selected as the desorption agent, and the desorption rate was 0.03 mL/min. After elution, distilled water was used to wash the regenerated column. The column adsorption experiment was repeated under the same conditions, and the regeneration efficiency (%) was defined as the ratio of the treatment capacity after each regeneration to that of the first adsorption experiment.

## 3. Results and Discussion

### 3.1. Adsorbent Characterization

#### 3.1.1. Cationic Polymer Content and FTIR Analysis

**Figure 1:**
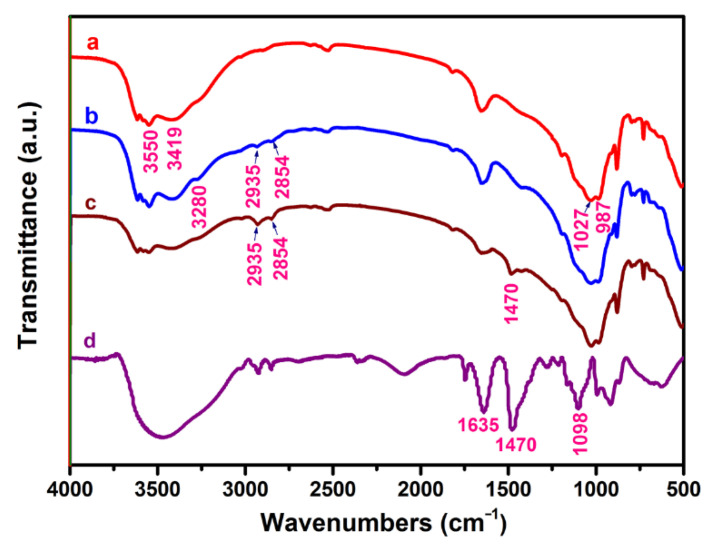
Fourier transform infrared spectroscopy (FTIR) spectra of samples. (**a**): attapulgite (ATP), (**b**): 3-aminopropyltriethoxysilane (APTES)-modified ATP (AM-ATP), (**c**): cation-modified ATP (CM-ATP), (**d**): polyepichlorohydrin-dimethylamine (EPI-DMA).

The content of EPI-DMA in CM-ATP was determined as 9.7 wt.%. The FTIR spectra are displayed in Figure 1. In the spectrum of ATP, there were multiple absorption peaks between 3400–3600 cm^−1^, which corresponded to the stretching vibration peaks of the (Mg)O-H and (Al)O-H bonds, and the hydroxyls of absorbed water. The absorption peak at 1027 cm^−1^ was the asymmetric stretching vibration peak of Si–O–Si (Al). For AM-ATP, the new peaks at 3280, 2935 and 2854 cm^−1^ were identified as the stretching vibrations of -NH_2_, methyl (–CH_3_) and methylene (–CH_2_), respectively. For EPI-DMA, the absorption peaks at 1635, 1470 and 1098 cm^−1^ indicated the presence of –CH_2_–N^+^R_3_-(quaternary ammonium group)-type nitrogen [29,32]. In the spectrum of CM-ATP, a new peak appeared at 1470 cm^−1^, which corresponded to the C-N. Additionally, the peaks assigned to methyl and methylene were further enhanced. These results indicated that the cationic polymer had been successfully grafted onto ATP.

#### 3.1.2. XRD Analysis 

**Figure 2:**
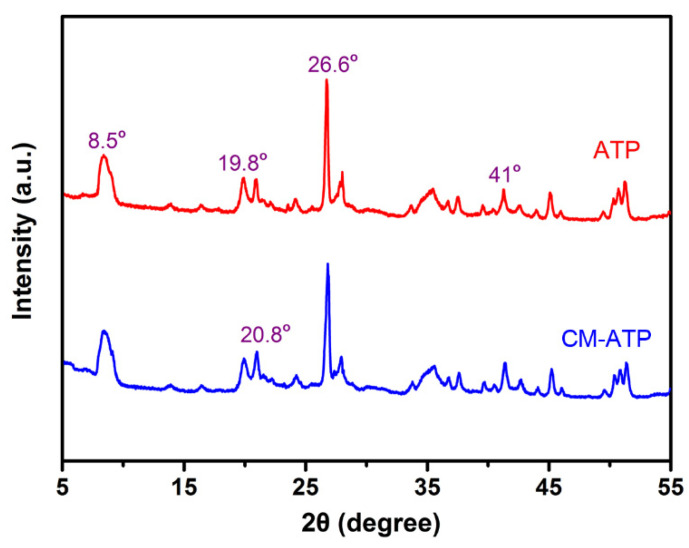
X-ray diffraction (XRD) patterns of samples.

Figure 2 shows the XRD patterns of ATP and CM-ATP. It was found that the reflections at 8.5°, 19.8° and 20.8° (2*θ* degree) were the characteristic diffraction peaks of ATP minerals. The strong diffraction peak at 26.6° was the characteristic peak of quartz. By comparing the patterns of CM-ATP and ATP, it could be seen that cationic modification did not change the original crystal structures of ATP. This verified that the cationic polymer was only grafted onto the surface of ATP, instead of the intercalation modification [19].

#### 3.1.3. SEM Analysis 

**Figure 3:**
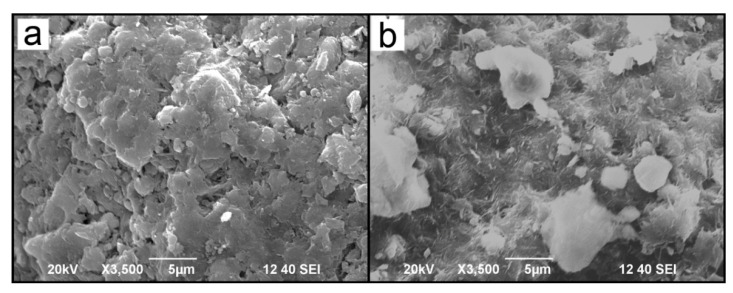
Scanning electron microscope (SEM) images of natural ATP (**a**) and CM-ATP (**b**).

From Figure 3: the natural ATP was shaped as a layered and laminated structure, which had pore canals and large spaces in or between the layers [33,34]. The CM-ATP had a distinct roughness, with less layers on the surface, and the pore canals in its layers decreased significantly. This could result from the cationic polymer EPI-DMA which had been grafted onto the ATP.

#### 3.1.4. Particle Size Distribution of Samples 

**Figure 4:**
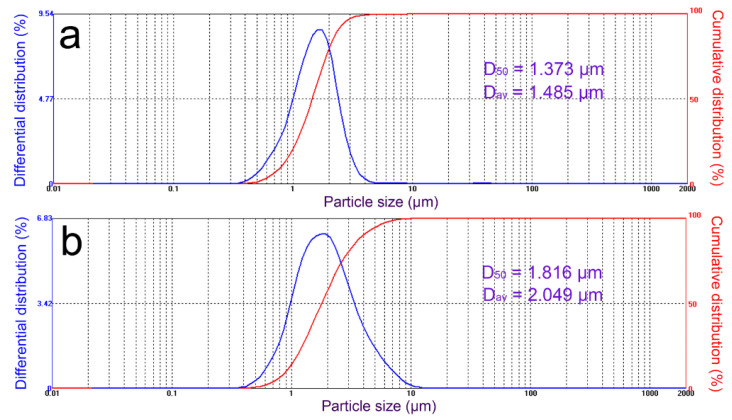
Particle size distribution of natural ATP (**a**) and CM-ATP (**b**). D_50_ and D_av_ are the median particle size and average particle size, respectively.

The particle size distribution of ATP and CM-ATP is shown in Figure 4. The particle size of ATP and CM-ATP were both normally distributed. The particle size distribution of CM-ATP was between 0.3 μm and 15 μm, and was slightly larger than that of ATP. The D_50_ and D_av_ of CM-ATP were both larger than the corresponding particle size of ATP. This might be due to a large amount of cationic polymer grafted onto the ATP, which promoted the mutual aggregation of particles. This could be confirmed using the SEM results (Figure 3).

#### 3.1.5. Specific Surface Area of Samples 

**Figure 5:**
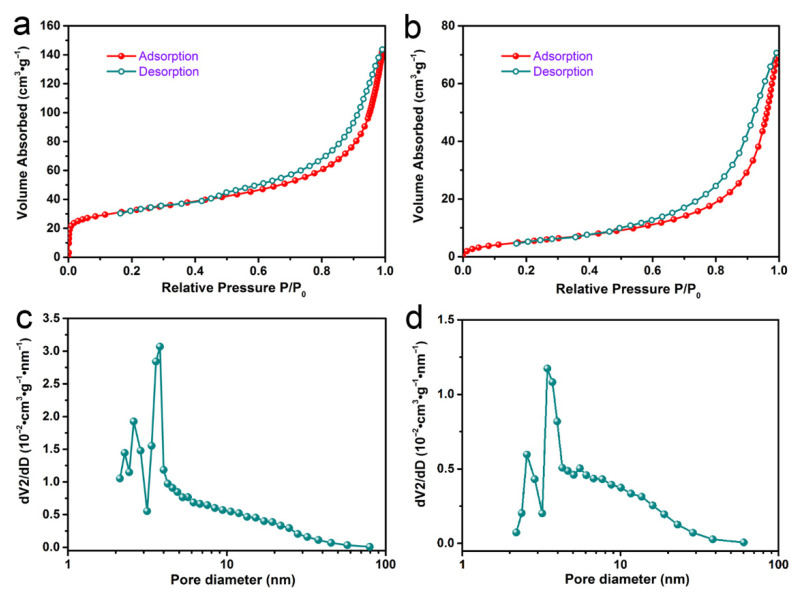
N_2_ adsorption-desorption isotherms and pore size distribution of ATP (**a**,**c**), and CM-ATP (**b**,**d**).

The N_2_ adsorption-desorption isotherms were utilized to analyze the surface area and pore size of samples, and the results are shown in Figure 5a,b. It could be concluded that both samples contained mesopore structures, as their N_2_ adsorption-desorption isotherms were characterized by type IV and had a hysteresis loop [35]. The BET-specific surface area of CM-ATP was less than that of ATP, which was similar to many other organic ATPs (see Table S3). In addition, the Barrett-Joyner-Halenda average mesoporous diameter of CM-ATP was larger than that of natural ATP (see Figure 5c,d; Table S3).

### 3.2. Dye Adsorption Studies

#### 3.2.1. Effect of Solution pH, Salt and Surfactant on Adsorption

**Figure 6:**
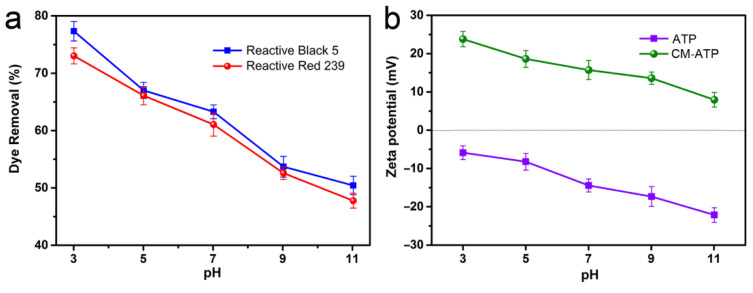
Effect of solution pH on dye removal of CM-ATP (**a**). The zeta potential values of ATP and CM-ATP (**b**).

The adsorption of dyes on CM-ATP (2 g/L) as a function of solution pH at 20 °C is shown in Figure 6a. The adsorption removal percentages of CM-ATP to RB5 and RR239 gradually decreased with the increase of solution pH. The reason might be the competition of excess negatively charged OH^−^ ions in solution with anionic dye molecules for adsorbing on CM-ATP [36].

The result could also be explained by the zeta potential analysis. As displayed in Figure 6b, it was evident that the maximum zeta potential value of CM-ATP was observed at pH 3, and the values gradually decreased with increasing pH. This result indicated that the electrostatic attraction between functional groups on CM-ATP and dye anions would decrease accordingly, resulting in lower adsorption capacity at higher solution pH. In addition, ATP has a negative surface charge in a wide pH range, which is also the main reason for its poor adsorption ability with respect to anionic dyes. Based on the results of the zeta potential analysis and the effect of solution pH on adsorption, it can be concluded that the main adsorption mechanism of CM-ATP to reactive dyes should be the electrostatic attraction between the cationic quaternary ammonium groups in the adsorbent and the anionic groups of dyes.

The effects of NaCl and DTAC on dye adsorption by CM-ATP are illustrated in Figure S1. From Figure S1a, the removal percentages of CM-ATP to RB5 and RR239 slightly increased with the increasing amount of NaCl in solution. The reason might be that the diffuse double layer on the surface of the adsorbent may have been compressed when the ionic strength of the solution increased; thus, the adsorption by electrostatic attraction was facilitated [37]. By contrast, the adsorption of the two dyes significantly declined with the presence of cationic surfactant DTAC, which might be ascribed to the formation of dye-surfactant aggregates [10].

#### 3.2.2. Adsorption Thermodynamics

The experimental data of CM-ATP (2 g/L) adsorbing dyes at different temperatures under neutral conditions are shown in Table S4, and the Gibbs free energy ∆G°, entropy change ∆S°, and enthalpy change ∆H° of the adsorption process were calculated and shown in Table 1. The ∆G° values were all negative under different temperature conditions, which meant the adsorption process of CM-ATP adsorbing dyes was spontaneous. The ∆H° values were all positive, indicating the endothermic process of reactive dyes adsorbed on CM-ATP; increasing the temperature could be beneficial to the adsorption. The ∆S° values of the entire adsorption system were positive, indicating that the disorder of the system was relatively increased during the adsorption process.

#### 3.2.3. Kinetic Studies

**Figure 7:**
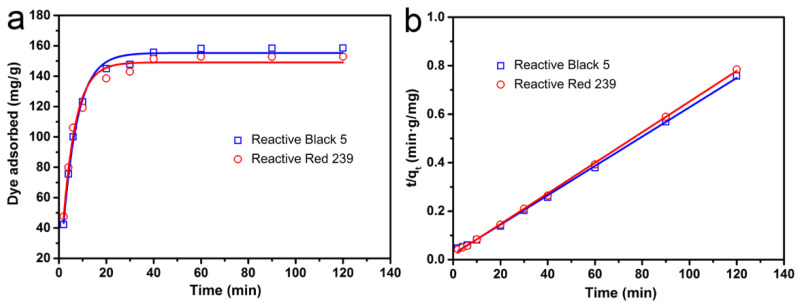
Kinetic adsorption fitting curves for dyes on CM-ATP. (**a**) pseudo-first-order kinetic model, (**b**) pseudo-second-order kinetic model.

The kinetic study is commonly used to estimate the adsorption rate and infer the reaction mechanism. The pseudo-first order and pseudo-second order kinetic models were evaluated, and the results are shown in Figure 7 and Table 2. Comparing the fitting results of the two kinetic models, it could be seen that the experimental data were significantly better in accord with the pseudo-second-order kinetic model. This result meant that the adsorption behavior of CM-ATP was chemical adsorption [38].

#### 3.2.4. Isotherm Studies

**Figure 8:**
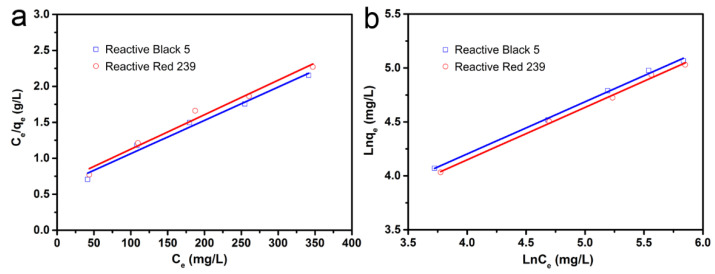
Langmuir (**a**) and Freundlich (**b**) adsorption isotherm plots.

Isotherm studies of the adsorption process were conducted by using the Langmuir and Freundlich isotherm models, and the results are shown in Figure 8 and Table 3. It was observed that the experimental data were better in accord with the Freundlich model; its correlation coefficients R^2^ for RB5 and RR239 were 0.996 and 0.995, respectively, higher than that of the Langmuir model. Based on the calculation results of the Langmuir model, the maximum adsorption capacities (*Q_0_*) of CM-ATP were 237.4 and 228.3 mg/g for RB5 and RR239, respectively. It can be seen from Table 4 that CM-ATP has relatively high adsorption capacities to reactive dyes, even compared with other adsorbents, indicating that this new type of adsorbent has potential practical application values.

### 3.3. Column Adsorption Studies

The typical breakthrough curves were determined by using ATP, AC and CM-ATP as column adsorbents for treating the RR239 solution (see Figure S2). From Figure S1, the breakthrough curve of each adsorbent showed a different excellent clear zone (100% removal [44]), in which the effluent was colorless. Based on the volumes of colorless effluent, the treatment capacities of adsorbents in the column adsorption process were calculated, and the results are given in Table 5. The treatment capacities of ATP significantly increased after cationic modification, and the treatment capacities of CM-ATP corresponding to RB5 and RR239 were 144.7 and 138.4 mg/g, respectively. This could be at least 80 times more than that of natural ATP and even three times greater than that of commercial activated carbon (AC). This result may be explained by the fact that grafted EPI-DMA will retard dye molecules passing through the column via electrostatic adsorption.

**Figure 9:**
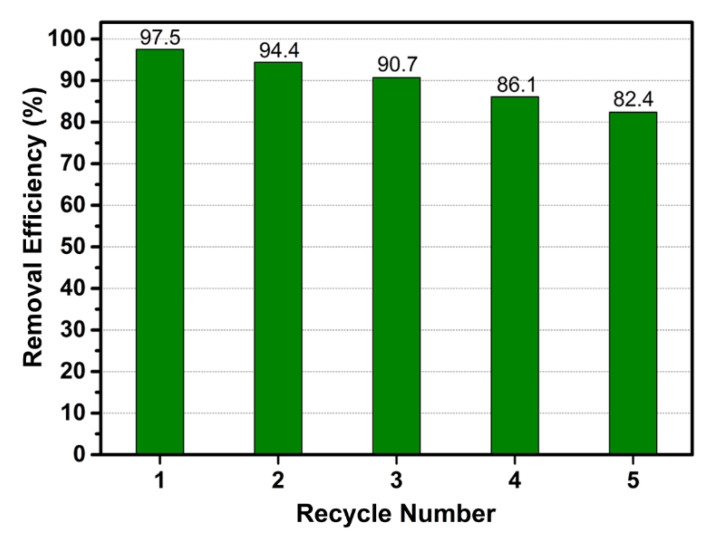
The regeneration efficiency of CM-ATP in column operation.

After column adsorption, the regeneration experiment was carried out by using dodecyl trimethyl ammonium chloride solution as the desorption agent. The results of the regeneration experiment are shown in Figure 9. CM-ATP had good stability and regeneration performance; regeneration efficiency still maintained over 80%, even after five successive desorption-adsorption cycles.

## 4. Conclusions

The cation-modified ATP was successfully synthesized by grafting the cationic polymer EPI-DMA onto natural ATP. The crystal structure of natural ATP wasn’t altered after cationic modification, but its specific surface area decreased. Equilibrium adsorption studies demonstrated that the maximum adsorption capacities of CM-ATP were 237.4 and 228.3 mg/g for RB5 and RR239, respectively. The adsorption processes were found to be endothermic, spontaneous, and influenced by solution pH and surfactant. The adsorption kinetics fitted the pseudo-second-order kinetic model. The column adsorption studies demonstrated that the fabricated adsorbent exhibited higher dye adsorption ability than both the natural ATP and AC, and could be used repeatedly. In conclusion, the cation-modified ATP has potential applications in the treatment of dye wastewaters in industry fields.

## Figures and Tables

**Table 1:** Thermodynamic parameters for the adsorption of reactive dyes on CM-ATP.

Dyes	∆H° (kJ∙mol^−1^)	∆S° (J∙mol^−1^∙K^−1^)	∆G° (kJ∙mol^−1^)				
			293 K	303 K	313 K	323 K	333 K
RB5	8.92	86.64	−16.48	−17.15	−18.32	−19.13	−19.81
RR239	9.03	86.30	−16.24	−17.02	−18.11	−18.93	−19.59

**Table 2:** The adsorption kinetics parameters of CM-ATP.

Dyes	*q_e,exp_* (mg·g^−1^)	Pseudo-First-Order			Pseudo-Second-Order		
		*q_e,cal_* (mg·g^−1^)	*k*_1_ (min^−1^)	R^2^	*q_e,cal_* (mg·g^−1^)	*k*_2_ (10^−2^ g·mg^−1^·min^−1^)	R^2^
RB5	158.5	155.1	0.163	0.981	159.8	0.158	0.998
RR239	152.9	148.2	0.187	0.976	154.5	0.195	0.999

**Table 3:** The isotherm adsorption parameters for dyes on CM-ATP.

Dyes	Langmuir			Freundlich		
	*Q*_0_ (mg/g)	K_L_ (10^−2^ L/mg)	R^2^	K_F_	n	R^2^
RB5	237.4	0.770	0.979	9.650	2.066	0.996
RR239	228.3	0.741	0.975	9.061	2.057	0.995

**Table 4:** Comparison of the adsorption of reactive dyes on CM-ATP and other alternative adsorbents.

Adsorbent	Experiment Conditions	Dyes	Q_0_ (mg/g)	Reference
CM-ATP	Solution pH: 7 Temperature: 20 °C Adsorbent mass: 2 g/L	RB5	237.4	This study
CM-ATP	Solution pH: 7 Temperature: 20 °C Adsorbent mass: 2 g/L	RR239	228.3	This study
Amino-functionalized attapulgite	Solution pH: natural initial pH Temperature: 20 °C Adsorbent mass: 3 g/L	Reactive Red 3BS	34.235	[19]
Sonication-surfactant-modified attapulgite	Solution pH: natural initial pH Temperature: 50 °C Adsorbent mass: 2 g/L	Reactive Red MF-3B	94.34	[18]
Cationic diatomite	Solution pH: 7 Temperature: 20 °C Adsorbent mass: 2 g/L	RB5	216.6	[39]
Powdered activated carbon	Solution pH: natural initial pH Temperature: 20 °C Adsorbent mass: -	RB5	58.823	[40]
Bone char	Solution pH: natural initial pH Temperature: 25 °C Adsorbent mass: 1 g/L	RB5	160	[41]
Magnetic chitosan	Solution pH: 5 Temperature: 25 °C Adsorbent mass: 1.75 g/L	Reactive Red 141	98.8	[42]
Weakly basic anion exchange resin (Amberlite IRA 67)	Solution pH: - Temperature: 25 °C Adsorbent mass: 10 g/L	RB5	66.4	[43]
Weakly basic anion exchange resin (Lewatit MonoPlus MP 62)	Solution pH: - Temperature: 25 °C Adsorbent mass: 10 g/L	RB5	796.1	[43]

**Table 5:** Sample treatment capacities for dye solutions in column adsorption process.

Dye Solutions	Treatment Capacity (mg/g)		
	ATP	AC	CM-ATP
RB5	1.8	47.1	144.7
RR239	1.7	44.3	138.4

## Data Availability

The data presented in this study are available on request from the corresponding author.

## Supplementary Materials

The following are available online at https://www.mdpi.com/1996-1944/14/4/792/s1, Table S1: Chemical structure of the reactive dyes; Table S2: Detailed information of all formulas and isotherms, kinetics models; Table S3: Surface area and pore size analyses of various modified ATP; Table S4: The effect of temperature on the adsorption of reactive dyes on CM-ATP; Scheme S1: Synthesis route of CM-ATP; Figure S1: Effects of NaCl and surfactant DTAC on dye adsorption by CM-ATP (2 g/L) with solution pH = 7; Figure S2: Breakthrough curves for dye adsorption in fixed-bed column.
