# Supplementary material for: Surface Modification of Attapulgite by Grafting Cationic Polymers for Treating Dye Wastewaters
Source: Materials (Basel). 2021 Feb 7;14(4):792. doi: 10.3390/ma14040792 (PMC7915886; doi:10.3390/ma14040792)
Supplement: Supplementary file 1 [file materials-14-00792-s001.pdf]

materials

Supplementary Material

Surface Modification of Attapulgite by Grafting Cationic Polymers for Treating Dye Wastewaters

Table S1. Chemical structure of the reactive dyes.

Dyes	Molecular structure
C.I. Reactive Black 5	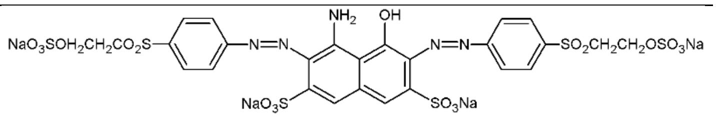
C.I. Reactive Red 239	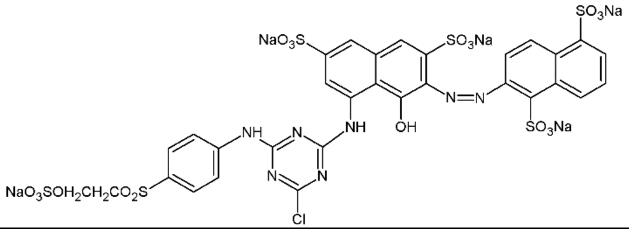

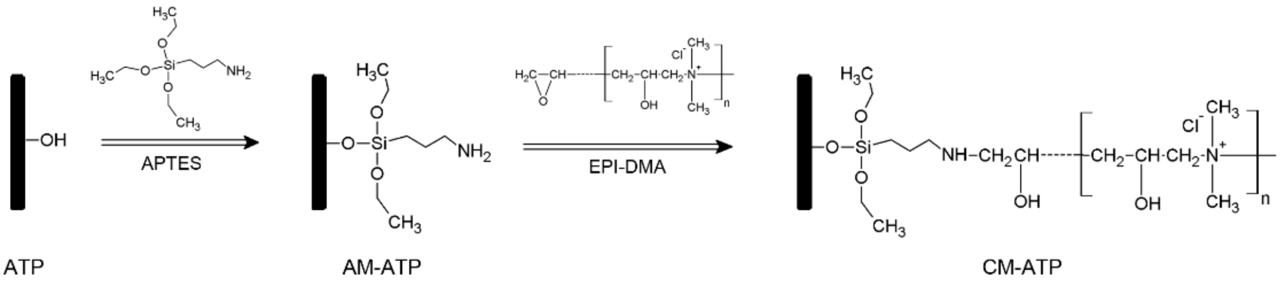

Scheme S1. Synthesis route of cation-modified attapulgite (CM-ATP).

Table S2. Detailed information on all formulas and isotherms, kinetics models.

Type	Model	Equation	Parameters
Formulas	Dye removal percentage	Dye removal (%) $= \frac{C_0 - C_1}{C_0} \times 100\%$	C_0 (mg/L): the initial dye concentration; C_1 (mg/L): the equilibrium dye concentration.
	Adsorption capacity	$q_e = \frac{V(C_0 - C_1)}{m}$	V (L): the dye solution volume; m (g): the adsorbent mass.
Kinetics models	Lagergren first-order	$q_t = q_e(1 - \exp(-k_1 t))$	q_e (mg/g): the adsorption capacity of the adsorbent at equilibrium; q_t (mg/g): the adsorption capacity of the adsorbent at time t; k_1 (1/min): the adsorption rate constant.
	Pseudo-second-order	$\frac{t}{q_t} = \frac{1}{k_2 q_e^2} + \frac{t}{q_e}$	q_e (mg/g): the adsorption capacity of the adsorbent at equilibrium; q_t (mg/g): the adsorption capacity of the adsorbent at time t; k_2 (g/mg·min): the rate constant

			determined by the plots of t/q_t versus t.
Isotherm models	Langmuir	$\frac{C_e}{q_e} = \frac{1}{Q_0 K_L} + \frac{C_e}{Q_0}$	C_e (mg/L): the equilibrium concentration of solutions; Q_e (mg/g): the adsorption capacity of the adsorbent; Q_m (mg/g): the maximum amount of adsorption corresponding to the monolayer coverage; K_L (L/mg) is the Langmuir constant related to the adsorption energy.
	Freundlich	$\ln q_e = \ln K_f + \frac{\ln C_e}{n}$	C_e (mg/L) is the equilibrium concentration of solutions; Q_e (mg/g) is the adsorption capacity of the adsorbent; K_F is the adsorption capacity in the unit concentration; 1/n is the intensity of adsorption.
Equations for calculating thermodynamics parameters		$\Delta G^0 = -RT \ln K$<br>$\ln K = -\frac{\Delta H^0}{RT} + \frac{\Delta S^0}{R}$<br>$K = \frac{q_e}{C_e}$	R (J·mol^{-1} ·K^{-1}), K (mL/g), T (K) and C_e (g/L) are the universal gas constant, equilibrium constant of the adsorption process, absolute solution temperature, and the equilibrium concentration of dye solution, respectively.

Table S3. Surface area and pore size analyses of various modified attapulgite (ATP).

Adsorbent	Surface area (m^2/g)	Average pore diameter (nm)	Reference
ATP	112.5	9.50	This study
CM-ATP	22.7	12.1	This study
ATP	62.409	-	[1]
Amino-functionalized attapulgite	152.66	-	[1]
ATP	167.24	-	[2]
Sonication-surfactant-modified attapulgite	63.96	-	[2]
ATP	136	-	[3]
Ethylenediamine-modified attapulgite	83	-	[3]

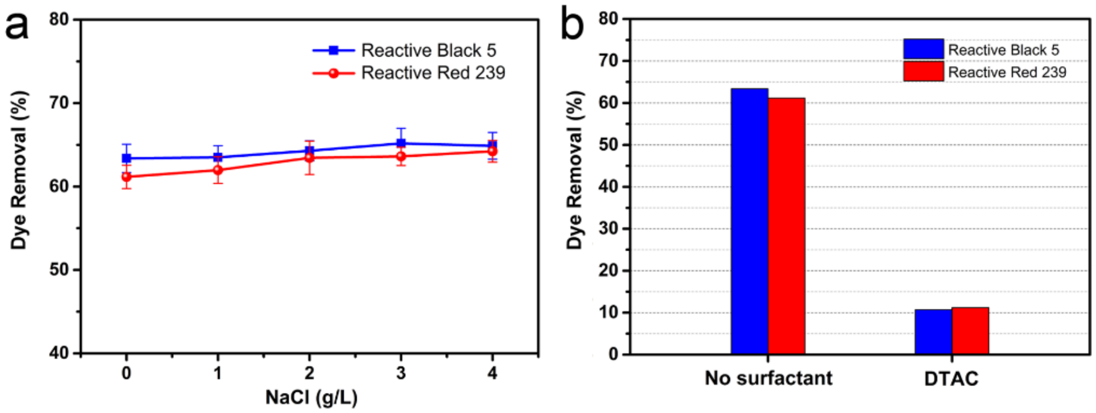

Figure S1. Effects of NaCl and surfactant dodecyltrimethylammonium chloride (DTAC) on dye adsorption by CM-ATP (2 g/L) with solution pH=7.

Table S4. The effect of temperature on the adsorption of reactive dyes by CM-ATP.

Dyes	Dye removal percentage (%)				
	**293 K**	**303 K**	**313 K**	**323 K**	**333 K**
RB5	63.38	64.4	69.5	71.28	71.9
RR239	61.16	63.2	67.8	69.7	70.3

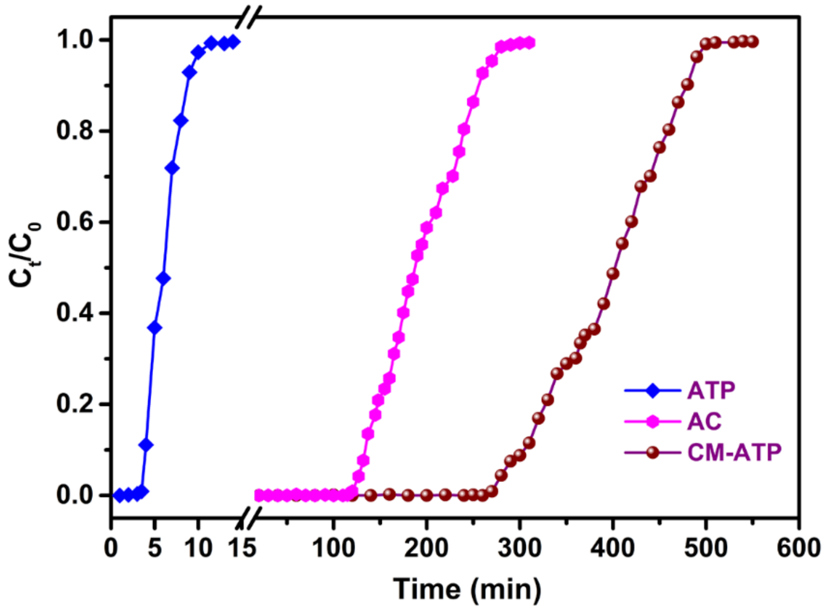

Figure S2. Breakthrough curves for dye adsorption in fixed-bed column. Experimental operating conditions: Reactive Red 239 solution (500 mg/L), flow rate = 2 mL/min, bed height = 2 cm.

References

1. Huang, J.; Liu, Y.; Jin, Q.; Wang, X.; Yang, J. Adsorption studies of a water soluble dye, Reactive Red MF-3B, using sonication-surfactant-modified attapulgite clay. *J. Hazard. Mater.* **2007**, *143*, 541-548.
2. Xue, A.; Zhou, S.; Zhao, Y.; Lu, X.; Han, P. Effective NH_2-grafting on attapulgite surfaces for adsorption of reactive dyes. *J. Hazard. Mater.* **2011**, *194*, 7-14.
3. Deng, Y.; Gao, Z.; Liu, B.; Hu, X.; Wei, Z.; Sun, C. Selective removal of lead from aqueous solutions by ethylenediamine-modified attapulgite. *Chem. Eng. J.* **2013**, *223*, 91-98.
